# Sequential Variational Autoencoder with Adversarial Classifier for Video Disentanglement

**DOI:** 10.3390/s23052515

**Published:** 2023-02-24

**Authors:** Takeshi Haga, Hiroshi Kera, Kazuhiko Kawamoto

**Affiliations:** 1 Department of Applied and Cognitive Informatics, Graduate School of Science and Engineering, Chiba University, Chiba 263-8522, Japan; 2 Graduate School of Engineering, Chiba University, Chiba 263-8522, Japan

**Keywords:** adversarial training, auxiliary adversarial classifier, inductive biases, sequential variational autoencoder, video disentanglement

## Abstract

In this paper, we propose a sequential variational autoencoder for video disentanglement, which is a representation learning method that can be used to separately extract static and dynamic features from videos. Building sequential variational autoencoders with a two-stream architecture induces inductive bias for video disentanglement. However, our preliminary experiment demonstrated that the two-stream architecture is insufficient for video disentanglement because static features frequently contain dynamic features. Additionally, we found that dynamic features are not discriminative in the latent space. To address these problems, we introduced an adversarial classifier using supervised learning into the two-stream architecture. The strong inductive bias through supervision separates dynamic features from static features and yields discriminative representations of the dynamic features. Through a comparison with other sequential variational autoencoders, we qualitatively and quantitatively demonstrate the effectiveness of the proposed method on the Sprites and MUG datasets.

## 1. Introduction

Representation learning, which involves the acquisition of low-dimensional latent variables from high-dimensional data such as videos and images, is an essential task in computer vision. Disentangling latent variables and associating one generative factor with one latent variable are objectives of representation learning. Such a disentangled representation can be helpful for downstream tasks, such as causal and controllable inferences, prediction, and generation [1].

Typical deep generative models for disentanglement include variational autoencoders (VAEs) [2] and generative adversarial nets (GANs) [3]. VAEs optimize the model and intermediate layers to learn the mapping between the input data and the generated results so that the relationship between the latent variables and the generated results can be interpreted. In contrast, GANs optimize only the conditional generators for disentanglement [4,5,6,7,8,9], without learning the mapping between them; that is, GANs fail to provide a direct relationship. Therefore, VAEs are preferred for optimizing the latent variables for disentanglement.

**Figure 1:**
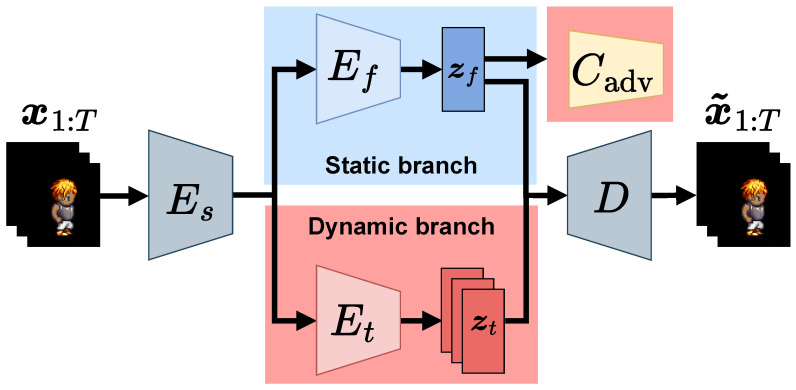
Overview of proposed model. The model consists of an auxiliary adversarial classifier $C_{\mathrm{adv}}$, a decoder *D*, a shared encoder $E_{s}$, a static encoder $E_{f}$, and a dynamic encoder $E_{t}$. $x_{1:T}$ and ${\tilde{x}}_{1:T}$ denote input and reconstructed videos, respectively, and $z_{f}$ and $z_{t}$ indicate static and dynamic latent variables, respectively.

Several studies have investigated image disentanglement using VAEs [10,11,12]. Locatello et al. [13] revealed that the basic VAE has difficulty obtaining disentangled representation without inductive biases. Therefore, subsequent studies developed model architectures and loss functions to strengthen inductive biases. Inductive biases for video disentanglement divide videos into dynamic (time-dependent) and static (time-independent) features. The disentangled sequential VAE (DSVAE) [14] uses an architecture-based inductive bias. The recent sequential VAEs are based on the two-stream architecture of DSVAE, as shown in Figure 1. DSVAE is the basic model for disentanglement [15,16,17] and sequential tasks [18,19,20].

**Figure 2:**
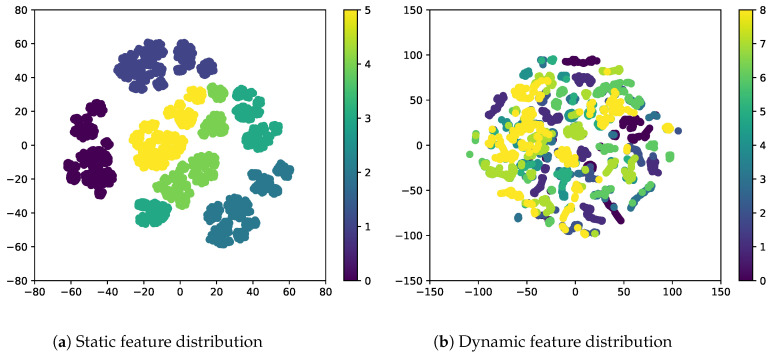
Disentangled sequential variational autoencoder (DSVAE): visualization of (**a**) static and (**b**) dynamic features in the latent spaces on the Sprites dataset using t-SNE [27]. The static features are separately distributed for each class, whereas the dynamic features overlap across the classes.

Nevertheless, Luo et al. [21] pointed out that DSVAE can collapse static features and ignore them in music audio. However, our preliminary experiments (Appendix A) showed that the static features in videos do not collapse but are sufficiently rich to represent videos because static features frequently include dynamic features. Therefore, dynamic features should be excluded from static features for video disentanglement. Moreover, static features are separately distributed for each class, whereas dynamic features overlap across classes, as shown in Figure 2. Therefore, the videos generated using dynamic features can contain a mix of several dynamic classes. These two problems can be attributed to the weak inductive bias of the two-stream architecture.

**Figure 3:**
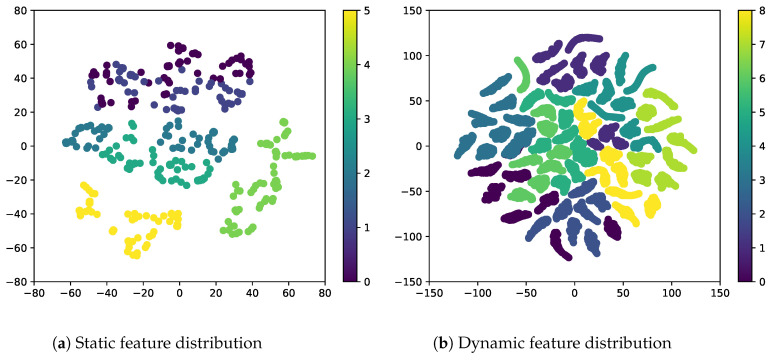
Ours: visualization of (**a**) static and (**b**) dynamic features in the latent spaces on the Sprites dataset using t-SNE [27]. The static and dynamic features are separately distributed for each class.

Adversarial classifiers are used for various applications, such as cross-domain image generation [22,23], face image classification [24], and speech generation [25]. Since the adversarial classifier uses supervision, it can yield a strong inductive bias for video disentanglement. The proposed sequential VAE has a two-stream architecture with an adversarial classifier $C_{\mathrm{adv}}$, as shown in Figure 1, to further strengthen the inductive bias. The blue and red regions denote the branches for static and dynamic features, respectively. The encoders $E_{s}$ and $E_{f}$ are trained to extract static feature $z_{f}$ from the static branch. As the adversarial classifier $C_{\mathrm{adv}}$ is trained to predict the labels $y_{t}$ of dynamic features $z_{1:T}=\left\{z_{1},\ldots ,z_{T}\right\}$ from static features $z_{f}$, the classifier $C_{\mathrm{adv}}$ is adversarial to the encoders $E_{s}$ and $E_{f}$. Adversarial learning excludes dynamic features from the static feature and yields a discriminative representation of the dynamic features, as shown in Figure 3, where the dynamic features are separately distributed for each class. Hence, the adversarial classifier can simultaneously solve the two problems in video disentanglement. We demonstrate the effectiveness of the proposed method on two benchmark video datasets, Sprites [14] and MUG [26], through quantitative and qualitative comparison with the state-of-art methods. The contributions of this study are summarized as follows:

- We identify the following two underlying problems caused by the basic two-stream VAE architecture for video disentanglement: (i) the static feature of a given video may include dynamic features; (ii) dynamic features are not discriminative in the latent space. To the best of our knowledge, no previous study has explored these problems.
- We propose a sequential VAE with an adversarial classifier to solve these two problems simultaneously. The supervision by an adversarial classifier provides a strong inductive bias for these problems. Moreover, we demonstrate that the adversarial classifier excludes dynamic features from the static feature, yielding discriminative dynamic features.

The remainder of this paper is organized as follows: Section 2 presents the related work on video disentanglement. Section 3 introduces the proposed method. The qualitative and quantitative results are presented and discussed in Section 4. Finally, the conclusions are presented in Section 5.

## 2. Related Work

VAEs are typically used to disentangle features. Earlier studies improved image disentanglement by adjusting the Kullback–Leibler (KL) divergence term for variational inference. Higgins et al. [10] introduced a weight coefficient to the KL divergence term so that the latent variable distribution approaches the standard normal distribution. Chen et al. [12] decomposed the KL divergence term and assigned a weight coefficient for each term to reduce reconstruction errors. However, Locatello et al. [13] found that inductive biases are crucial for disentanglement. Therefore, subsequent studies developed model architectures and loss functions to strengthen inductive biases.

Inductive biases divide videos into static and dynamic features. Typical VAEs use deep recurrent models to disentangle the sequential data. Chung et al. [28] incorporated VAE into a long short-term memory (LSTM) model [29]. Hsu et al. [30] incorporated a hierarchical structure into VAE. As the hierarchical structure can separate identity information (e.g., the speech of a speaker) from environmental information (e.g., noise), the model improves the audio data generation quality. DSVAE [14] uses a two-stream architecture-based inductive bias, as shown in Figure 1. The architecture has two branches that separately extract static and dynamic features from videos. DSVAE is used for disentanglement and sequential tasks, such as domain generalization [18], zero-shot generation [19], and time prediction [20].

Zhu et al. [15] proposed a self-supervised sequential VAE (S3VAE) based on the DSVAE architecture. The S3VAE introduces additional loss terms related to each latent variable based on the mutual information between the input and latent variables. S3VAE uses pre-trained optical flow [31] or landmark detectors [32] to compute the regularization term. Therefore, the overall performance of S3VAE depends on the performance of off-the-shelf detectors for given datasets. According to Han et al. [16], the KL divergence used to measure the distance between the prior and posterior distributions is too restrictive. Hence, they proposed the disentangled recurrent Wasserstein autoencoder (R-WAE) [16], using the Wasserstein distance. The Wasserstein distance uses maximum mean discrepancy (MMD) or GANs, whose hyperparameters are known to be difficult to turn [17]. Bai et al. [17] proposed the contrastively DSVAE (C-DSVAE) method that strengthens the inductive bias using contrastive learning with an expensive self-supervised data augmentation. Since contrastive learning does not use supervision on labels, C-DSVAE does not directly solve the overlapping problem, as shown in Figure 2. Mathieu et al. [33] and Ding et al. [34] used an auxiliary adversarial classifier to encourage the disentanglement of latent variables in images. The above approaches are similar to our approach, but they do not adopt the two-stream VAE architecture, limiting them to image disentanglement applications. Moreover, the overlapping problem in the dynamic latent space is not addressed.

**Figure 4:**
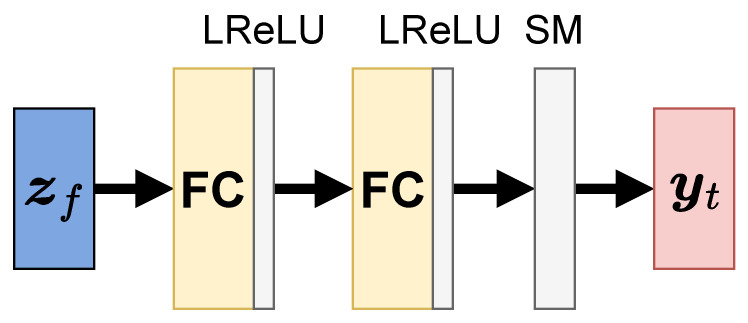
Overview of the auxiliary adversarial classifier $C_{\mathrm{adv}}$. The classifier consists of MLP with two fully connected (FC) layers and leaky ReLU (LReLU) and a softmax (SM) layer. The output is the predicted distribution $y_{t}$ of class labels for dynamic features.

In this study, we incorporated an adversarial classifier [22] into the static branch of the two-stream architecture, as shown in Figure 1. The supervision of dynamic labels by the adversarial classifier provides a strong inductive bias for video disentanglement. Furthermore, the overlapping problem in the dynamic latent space is solved in the supervised learning framework. The existing sequential VAEs do not address this problem or provide a direct means of solving it. Additionally, the proposed method requires no auxiliary optical flow detectors for disentanglement, and the adversarial classifier is easily learnable because of the simple architecture, as shown in Figure 4.

## 3. Method

We propose a two-stream sequential VAE with an adversarial classifier for video disentanglement, as shown in Figure 1. The classifier is trained, as shown in Figure 4, to avoid extracting dynamic features from the static branch of the two-stream sequential VAE and obtain discriminative representations of dynamic features in the latent space, as shown in Figure 3.

### 3.1. Variational Inference

The sequential VAE encodes sequential data, such as video or audio, into latent variables $z\in R^{M_{z}}$ and factorizes z into two disentangled variables: static variable $z_{f}\in R^{M_{f}}$ and dynamic variable $z_{t}\in R^{M_{t}}$, where $M_{z}=M_{f}+M_{t}$. The dynamic variable represents those features that change dynamically over time, whereas the static variable represents a constant feature. We assume that the probability distribution on z is factorized as follows:

(1) $$p\left(z\right)=p\left(z_{f}\right)p\left(z_{1:T}\right)=p\left(z_{f}\right)\prod_{t=1}^{T}p\left(z_{t}|z_{<t}\right),$$

where $z_{1:T}=\left\{z_{1},\ldots ,z_{T}\right\}$ and $z_{<t}=\left\{z_{1},\ldots ,z_{t-1}\right\}$. We assume the priors $p(z_{f})$ and $p(z_{t}|z_{<t})$ are the standard normal and normal distributions, respectively, that is,

(2) $$\begin{array}{cc} p(z_{f}) & =N(0,I) \end{array}$$

(3) $$\begin{array}{cc} p(z_{t}|z_{<t}) & =N(\mu_{t},\sigma_{t}^{2}I), \end{array}$$

where N(μ,Σ) denotes the multivariate normal distribution with a mean vector μ and covariance matrix Σ, and I denotes an identity matrix of size $M_{f}$ or $M_{t}$. Parameters $\mu_{t}$ and $\sigma_{t}^{2}$ are conditioned on all previous dynamic variables $z_{<t}$. The sequential VAEs compute $\mu_{t}$ and $\sigma_{t}^{2}$ using LSTM. We assume our inference model is obtained as follows:

(4) $$q\left(z|x_{1:T}\right)=q\left(z_{f},z_{t}|x_{1:T}\right)=q\left(z_{f}|x_{1:T}\right)\prod_{t=1}^{T}q\left(z_{t}|x_{<t}\right),$$

where $x_{1:T}=\left\{x_{1},\ldots ,x_{T}\right\}$ and $x_{t}\in R^{M_{x}}$ denote a frame at time *t* of an input video of length *T*. The loss function for variational inference is expressed as follows:

(5) $$\begin{array}{cc} L_{\mathrm{vae}}\left(x_{1:T},z_{f},z_{1:T}\right)= & E_{q(z_{1:T},z_{f}|x_{1:T})}\left[-\sum_{t=1}^{T}\log p\left(x_{t}|z_{f},z_{t}\right)\right]\\ \; & +D_{\mathrm{KL}}\left[q\left(z_{f}|x_{1:T}\right)∥p\left(z_{f}\right)\right]+\sum_{t=1}^{T}D_{\mathrm{KL}}\left[q\left(z_{t}|x_{\leq t}\right)∥p\left(z_{t}|z_{<t}\right)\right], \end{array}$$

where $D_{\mathrm{KL}}\left(q|p\right)$ denotes the KL divergence between the probability distributions *q* and *p*. The weights of the decoder *D* and dynamic encoder $E_{t}$ in Figure 1 are minimized based on the gradient descent using the loss function in Equation (5).

### 3.2. Auxiliary Adversarial Classifier

We introduce the auxiliary adversarial classifier $C_{\mathrm{adv}}$ to strengthen the inductive bias for video disentanglement. Our preliminary experiments (Appendix A) reveal that the static branch extracts static and dynamic features in the input videos. To avoid extracting dynamic features in the static branch, we adversarially train the classifier $C_{\mathrm{adv}}$, positioned after the static branch, as shown in Figure 1. Moreover, the adversarial classifier $C_{\mathrm{adv}}$ provides discriminative representations of dynamic features in the latent space, as shown in Figure 3.

The detailed architecture of the adversarial classifier $C_{\mathrm{adv}}$ is shown in Figure 4. The classifier $C_{\mathrm{adv}}$ is trained to predict the dynamic feature labels from the static features $z_{f}$. The network consists of a multilayer perceptron (MLP) with two fully-connected layers and a leaky rectified linear unit (LReLU). The predicted labels are output through the softmax layer.

The loss function for $C_{\mathrm{adv}}$ is a cross-entropy loss, expressed as follows:

(6) $$L_{C_{\mathrm{adv}}}\left(z_{f};l_{t}\right)=\sum_{j=1}^{K}-l_{t}^{j}\log y_{t}^{j},$$

where $l_{t}=\left(l_{t}^{1},l_{t}^{2},\ldots ,l_{t}^{K}\right)$ denotes a one-hot label vector for dynamic features and $y_{t}=\left(y_{t}^{1},y_{t}^{2},\ldots ,y_{t}^{K}\right)$ denotes the predictive distribution of the classifier $C_{\mathrm{adv}}$, expressed as follows:

(7) $$y_{t}=C_{\mathrm{adv}}\left(z_{f}\right)=\mathrm{softmax}(Wz_{f}),$$

where $z_{f}$ and W denote the static branch output and MLP weight, respectively. Since the static branch is trained to extract static features, the classifier $C_{\mathrm{adv}}$ is adversarial to encoder *E*. For adversarial training, we reverse the gradient of the loss in Equation (6) as follows:

(8) $$L_{E}^{\mathrm{adv}}\left(z_{f};l_{t}\right)=-L_{C_{\mathrm{adv}}}\left(z_{f};l_{t}\right)=\sum_{j=1}^{K}l_{t}^{j}\log y_{t}^{j},$$

where $L_{E}^{\mathrm{adv}}$ denotes the adversarial term of encoder *E*.

In summary, we minimize the total loss function of the VAE loss in Equation (5) and gradient reversal loss in Equation (8) as follows:

(9) $$L_{\mathrm{vae}}\left(x_{1:T},z_{f},z_{1:T}\right)+\beta_{E}L_{E}^{\mathrm{adv}}\left(z_{f};l_{t}\right)⟶\min$$

where $\beta_{E}$ denotes the weight coefficient of the adversarial term; we set $\beta_{E}=10$ in the experiments. The above training algorithm is given by Algorithm 1.
**Algorithm 1** Training for sequential VAE with adversarial classifier.**Require:***X*: Set of videos with *T* frames
**Require:**$l_{t}$: Class labels of dynamic features
**Require:**$\beta_{E}$: Weight coefficient of adversarial term $L_{E}^{\mathrm{adv}}$**Require:**$\theta_{D}$: Parameters of decoder *D***Require:**$\theta_{E_{s}},\theta_{E_{f}},\theta_{E_{t}}$: Parameters of encoders $E_{s}$ (shared), $E_{f}$ (static), and $E_{t}$ (dynamic)
**Require:**$\theta_{C_{\mathrm{adv}}}$: Parameters of adversarial classifier $C_{\mathrm{adv}}$ 1: **while**
$\theta_{D},\theta_{E_{s}},\theta_{E_{f}},\theta_{E_{t}},\theta_{C_{\mathrm{adv}}}$ not converged **do** 2:    Sample $x_{1:T}$ from *X* 3:    Encode static variable $z_{f}=E_{f}\left(E_{s}\left(x_{1}\right),\ldots ,E_{s}\left(x_{T}\right)\right)$ 4:     **for** t=1 to *T*  **do** 5:             Encode dynamic variable $z_{t}=E_{t}\left(E_{s}\left(x_{1}\right),\ldots ,E_{s}\left(x_{t}\right)\right)$ 6:     **end for** 7:    Calculate gradients of losses in Equations (5), (6) and (8). 8:            $g_{D}\leftarrow \nabla_{\theta_{D}}L_{\mathrm{vae}}\left(x_{1:T},z_{f},z_{1:T}\right)$ 9:            $g_{E_{s}}\leftarrow \nabla_{\theta_{E_{s}}}\left(L_{\mathrm{vae}}\left(x_{1:T},z_{f},z_{1:T}\right)+\beta_{E}L_{E}^{\mathrm{adv}}\left(z_{f};l_{t}\right)\right)$ 10:          $g_{E_{f}}\leftarrow \nabla_{\theta_{E_{f}}}\left(L_{\mathrm{vae}}\left(x_{1:T},z_{f},z_{1:T}\right)+\beta_{E}L_{E}^{\mathrm{adv}}\left(z_{f};l_{t}\right)\right)$ 11:          $g_{E_{t}}\leftarrow \nabla_{\theta_{E_{t}}}L_{\mathrm{vae}}\left(x_{1:T},z_{f},z_{1:T}\right)$ 12:          $g_{C_{\mathrm{adv}}}\leftarrow \nabla_{\theta_{C_{\mathrm{adv}}}}L_{C_{\mathrm{adv}}}\left(z_{f};l_{t}\right)$ 13:    Update parameters $\theta_{D},\theta_{E_{s}},\theta_{E_{f}},\theta_{E_{t}},\theta_{C_{\mathrm{adv}}}$ with the gradients using Adam 14: **end while** 15: **return**
$\theta_{D},\theta_{E_{s}},\theta_{E_{f}},\theta_{E_{t}},\theta_{C_{\mathrm{adv}}}$ (Resulting parameters)

## 4. Experiments

### 4.1. Datasets

**Figure 5:**
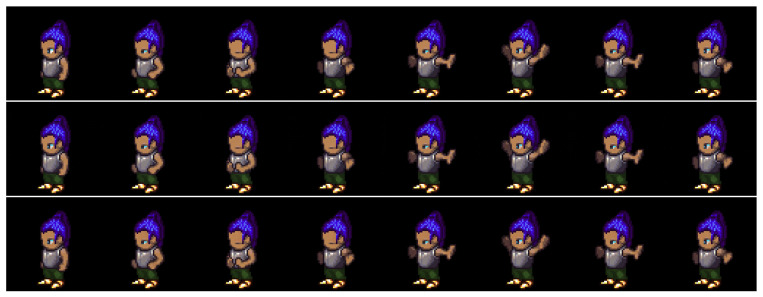
Example of reconstruction on the Sprites dataset. The top, middle, and bottom rows denote the input video and results using the DSVAE and proposed methods, respectively.

We evaluated the effectiveness of the auxiliary adversarial classifiers on the Sprites [14] and MUG [26] video datasets. The Sprites dataset consists of video game character animations, as shown in Figure 5. We generated 1296 character appearances by changing hairstyles, tops, skin, and pants. For each character, we generated three character motions from three viewpoints: walking, spellcasting, and slashing. Thus, the total motion category was nine. We sampled 1000 animations for training data and 296 animations for test data. The data consisted of eight frames of size 64×64. The MUG [26] dataset contains videos of the real-world facial expressions of 52 subjects. Each subject performs six facial expressions: anger, fear, disgust, happiness, sadness, and surprise. We split the dataset into 75% training and 25% testing, as suggested in [15,17]. The data consisted of 15 frames of size 64×64. Table 1 and Table 2 list the training conditions, and Appendix B shows the model architecture of the trained model.

### 4.2. Qualitative Results

#### 4.2.1. Reconstruction

We compared the proposed method with DSVAE [14] using a reconstruction task experiment in which a video was generated identically to the given input video. With this experiment, we examined whether the adversarial classifier degrades the generative ability of DSVAE; that is, this task was not intended to evaluate disentanglement performance.

**Figure 6:**
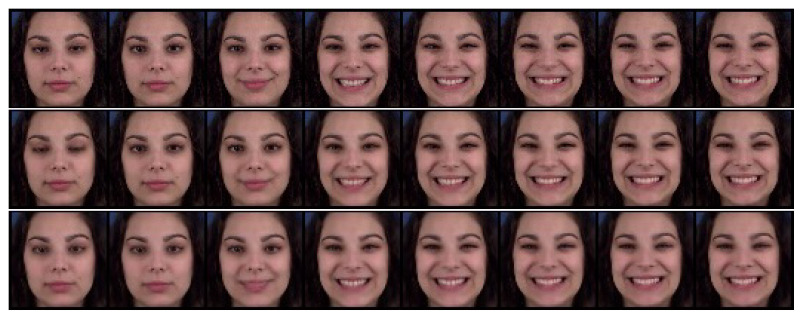
Example of reconstruction on the MUG dataset. The top, middle, and bottom rows denote the input video and the results using DSVAE and the proposed method, respectively.

Figure 5 and Figure 6 show examples of the generated results for the Sprites [14] and MUG [26] datasets, respectively. The top, middle, and bottom rows show the input video and the results using the DSVAE [14] and the proposed method, respectively. We edited the generated results for the MUG dataset into eight frames to fit the margin of the paper. Both methods provide videos identical to the input videos on both datasets. Moreover, the root-mean-squared error (RMSE) between the input and reconstructed videos per pixel was calculated. The pixel values in the RGB color space were normalized to [0,1]. The results in Table 3 reveal that the differences between their RMSEs are 0.000 and 0.003 for the Sprites and MUG datasets, respectively. Similar to the results shown in the figures, the differences are perceptually negligible.

#### 4.2.2. Zero-Replaced Video Generation

In this experiment, we investigated whether the adversarial classifier can solve the overlapping problem in dynamic features, as shown in Figure 2. As shown in Figure 3, the adversarial classifier separately distributes the overlapped features for each class, thereby yielding discriminative representations. Here, for the given videos, we created zero-replaced videos, generated by fixing the dynamic features and replacing the static feature with zero vectors. If the dynamic features were discriminative, a video identical to the input video would be generated.

**Figure 7:**
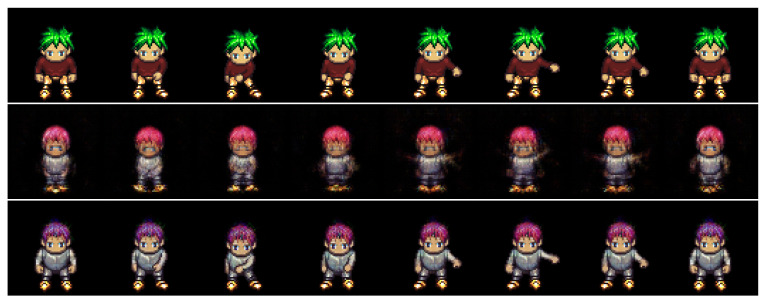
Example of zero-replaced generation on the Sprites dataset. We fixed the dynamic variables and replaced the static variable with zero. The top, middle, and bottom rows denote the input video and the results using DSVAE and the proposed method, respectively.

**Figure 8:**
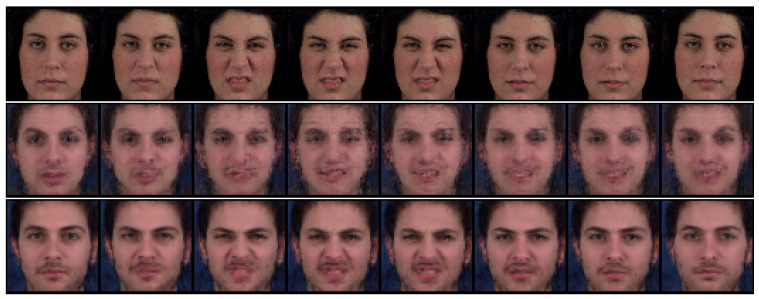
Example of zero-replaced generation on the MUG dataset. We fixed the dynamic variables and replaced the static variable with zero. The top, middle, and bottom rows denote the input video and the results using DSVAE and the proposed method, respectively.

Figure 7 and Figure 8 show the generated examples videos for the Sprites and MUG datasets, respectively. The top, middle, and bottom rows denote the input video and the results using DSVAE and the proposed method, respectively. As shown in Figure 7 and Figure 8, DSVAE generated a blur video with a different motion from the input video, although the dynamic features were fixed. Conversely, the proposed method generated a clear video similar to the input video. Therefore, the adversarial classifier disentangled the video into static and dynamic features, solving the overlapping problem simultaneously.

#### 4.2.3. Random Video Generation

In this experiment, we evaluated the disentanglement performance of the proposed method using randomly generated videos. The procedure for random video generation was as follows: First, we input a video from the Sprites or MUG dataset into the sequential VAE and extracted static and dynamic features. Next, we fixed one of the two features and replaced the other feature with a randomly generated feature from the prior distribution. The sequential VAE decoder generated the random video by concatenating the two features. If the sequential VAE disentangled the random video into static and dynamic features, we obtained the video conditioned on fixed and randomly generated features.

**Figure 9:**
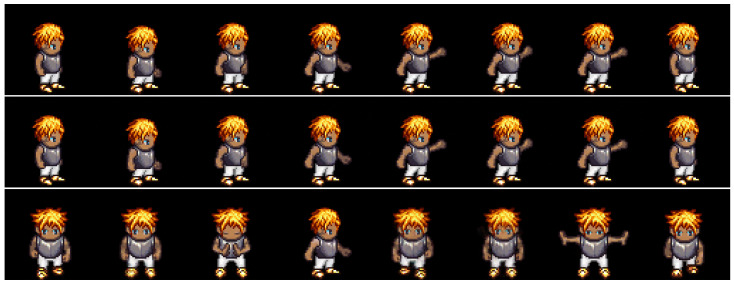
Example of randomly sampled generation on the Sprites dataset. We fixed the static variable and randomly sampled the dynamic variables. The top, middle, and bottom rows denote the input video and the results using DSVAE and the proposed method, respectively.

**Figure 10:**
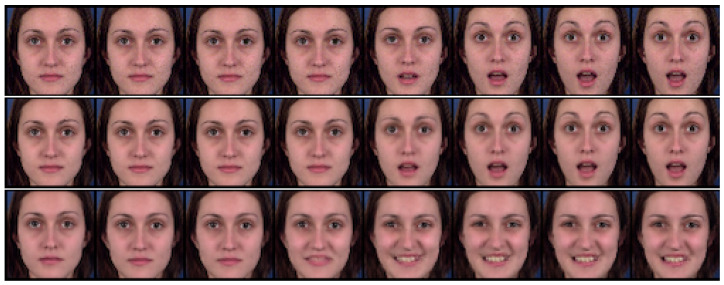
Example of randomly sampled generation on the MUG dataset. We fixed the static variable and randomly sampled the dynamic variables. The top, middle, and bottom rows denote the input video and the results using DSVAE and the proposed method, respectively.

Figure 9 and Figure 10 show randomly generated examples for the Sprites and MUG datasets, respectively. We generated these examples by fixing the static feature $z_{f}$ and randomly sampling the dynamic feature $z_{t}$ from the prior distribution $p(z_{t}|z_{<t})$. The top, middle, and bottom rows denote the input video and the results using DSVAE and the proposed method, respectively. Although we randomly sampled the dynamic features, DSVAE generated similar videos as the input videos. Conversely, the proposed method generated videos with randomly sampled motions, maintaining the static classes of the input videos. Therefore, the proposed method outperformed DSVAE.

**Figure 11:**
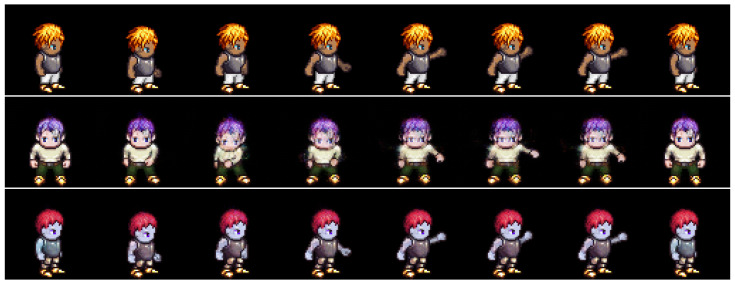
Example of randomly sampled generation on the Sprites dataset. We fixed the dynamic variables and randomly sampled the static variable. The top, middle, and bottom rows denote the input video and the results using DSVAE and the proposed method, respectively.

**Figure 12:**
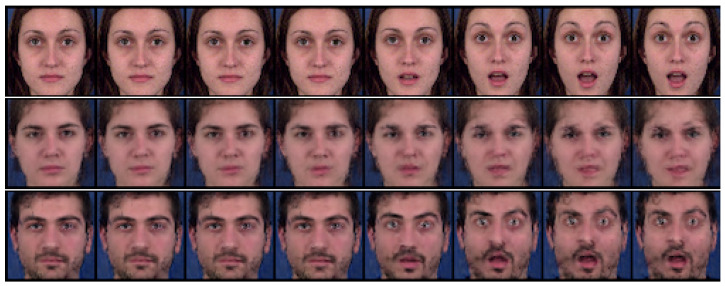
Example of randomly sampled generation on the MUG dataset. We fixed the dynamic variables and randomly sampled the static variable. The top, middle, and bottom rows denote the input video and the results using DSVAE and the proposed method, respectively.

We next generated video examples by randomly sampling the static feature from the prior distribution $p(z_{f})$ and fixing the dynamic features $z_{t}$. Figure 11 and Figure 12 show the generated examples on the Sprites and MUG datasets, respectively. The top, middle, and bottom rows denote the input video and the results using DSVAE and the proposed method, respectively. Although the dynamic features were fixed, DSVAE generated videos with different characters and motions than the input videos. Conversely, the proposed method only changed the character or person, and the generated videos were clear.

We summarize the experimental results as follows: Because DSVAE did not disentangle the videos into static and dynamic features, the static features partly included dynamic features. In contrast, the proposed method outperformed DSVAE in terms of disentanglement performance and video quality.

### 4.3. Quantitative Results

We compared the proposed method with four sequential VAEs: DSVAE [14], S3VAE [15], R-WAE [16], and C-DSVAE [17]. We obtained the quantitative results of the existing methods from the literature and omitted them when not mentioned. For C-DSVAE [17], we reproduced the results using a source code with its hyperparameters provided by the authors. For quantitative evaluation, we used the following metrics: classification accuracy, inception score, intra-entropy, and inter-entropy. For entropy-based metrics, see Appendix C. We reported the mean and standard deviation of the accuracy of the proposed method based on ten evaluations.

#### 4.3.1. Classification Accuracy

A classifier determines whether each sequential VAE generates the desired videos. Similar to [17], we prepared a classifier with five convolutional layers and trained it using the train and test data.

As explained in Section 4.2.3, we produced several videos through random video generation, which involves fixing either static or dynamic features and randomly sampling the other. Here, if features are disentangled, the accuracy of the class with fixed features should be high, whereas that of the randomly sampled features should achieve random accuracy. The random accuracyis derived as follows: $p_{k}$, k=1,…,K denotes the probability that a latent variable sampled from the prior distribution is class *k*. If input videos are uniformly sampled, the class probability is 1/K for all classes. Therefore, the probability of identical class pairs, i.e., the probability of correct prediction, is $\left(1/K\right)\sum_{k=1}^{K}p_{k}=1/K$. For the Sprites dataset, the random accuracies for the static and dynamic classes were 16.7% (≈1/6) and 11.1% (≈1/9), respectively, because the classes associated with the static and dynamic features were six and nine, respectively. For the MUG dataset, the random accuracies of the static and dynamic classes were 1.92% (≈1/52) and 16.7% (≈1/6), respectively, because the classes associated with the static and dynamic features were 52 and 6, respectively.

Table 4 shows the classification accuracy for the videos generated using fixed static features and randomly sampled dynamic features. For the Sprites dataset, as shown in Table 4a, DSVAE and the proposed method provided 100.0% and 99.0% accuracies for the static classes, respectively. Moreover, the proposed method provided an accuracy of 11.3%, which was closer to the random accuracy than the 11.1% for the dynamic classes. The MUG results were similar to those on the Sprites dataset. However, as shown in Table 4b, the proposed method provided an accuracy of 21.1%, which was closer to the random accuracy than the 16.7% for dynamic classes. For the Sprites and MUG datasets, the accuracy of DSVAE was close to 100% for the dynamic classes. This implied that changing the dynamic features did not affect the generated videos. Therefore, DSVAE could not disentangle the static and dynamic features of the videos. We only compared the proposed method with DSVAE in Table 4 because we implemented DSVAE on our own.

In contrast, Table 5 displays the classification accuracy for the videos generated using randomly sampled static features and fixed dynamic features. For the Sprite dataset, Table 5a demonstrates that the proposed method achieved 100% accuracy for dynamic classes and 16.47% for static classes, close to the random accuracy (16.67%). These accuracies were comparable to those of C-DSVAE results reproduced using the source code provided by the authors. For the MUG dataset, Table 5b demonstrates that C-DSVAE provided the highest accuracy of 81.16% for the fixed dynamic classes, followed by the proposed method with 77.52%. However, our reproduced C-DSVAE provided a 47.03% accuracy, which was a significant drop. We used a source code with its hyperparameters provided by the authors for the MUG dataset, but C-DSVAE required delicate tuning. In contrast, the proposed method consistently provided higher accuracy over the ten runs. The accuracy of the proposed method for static classes was 2.85%, which was also close to the random accuracy (1.92%).

#### 4.3.2. Entropy-Based Metrics

We evaluated disentanglement performance using three entropy-based metrics: inception score (IS), intra-entropy, H(y|x), and inter-entropy H(y) (Appendix C). For evaluation, we generated test videos to ensure uniform class distribution. We used the classifier in Section 4.3.1 for computing the entropy-based metrics.

Table 6a demonstrates the results for the Sprites dataset. The proposed method provided the best performance in the three metrics. Table 6b demonstrates the MUG results. For IS and H(y|x), C-DSVAE provided the highest accuracy of 5.341 and 0.092, respectively. However, our reproduced C-DSVAE degraded the performance, scoring 2.362 and 0.855 for IS and H(y|x), respectively. In contrast, the proposed method provided the best H(y) and worst H(y|x) and IS because of the unsatisfactory detail quality of the generated videos. In Table 5b, the accuracy for the dynamic classes using the proposed method was 77.52%. However, Figure 12 demonstrates that the details were blurry. Therefore, H(y|x), an index of realism, was higher, and the inception score IS, an index of the overall evaluation, was lower than other previous methods. To improve the quality of the generated results, a hierarchical architecture or a discriminator may be added, which will be considered in the future.

## 5. Conclusions

In this study, we introduced an auxiliary adversarial classifier into the sequential VAE to strengthen the induction bias for video disentanglement. With the basic two-stream VAE architecture, static features tend to include dynamic features, which are not discriminative in the latent space. Our experiments demonstrated that the proposed method with the adversarial classifier could simultaneously solve these problems. The proposed method also outperformed the previous studies in three evaluation metrics.

The proposed method provides a strong inductive bias for video disentanglement using supervised learning, that is, it requires class labels to learn the adversarial classifier.

Therefore, the proposed method cannot be used with data that lack random behaviors or class labels for dynamic features. The adversarial classifier needs only class labels for dynamic features, not static ones. In this sense, our approach can be categorized as weakly supervised learning. To mitigate weakly supervised learning, two possible directions may be taken for future work. One is adopting a self-supervised learning technique such as pseudo-labeling. The other is using semi-supervised learning by utilizing a combination of labeled and unlabeled data for training. These techniques reduce the labeling burden while retaining the strong inductive bias through supervision for application in various downstream tasks.

## Figures and Tables

**Table 1:** Learning condition for the Sprites dataset.

Appearance	1296 (4 parts × 6 categories)
	(train = 1000, test = 296)
Motion	9 (3ways×3directions)
Format	(channel, frame, height, weight) = (3, 8, 64, 64)
Batch size	100
Training Epochs	1000
Optimization	Adam (α=0.001, $\beta_{1}=0.9$, $\beta_{2}=0.999$)
$z_{t}$ Dimension	32
$z_{f}$ Dimension	256

**Table 2:** Learning condition for the MUG dataset.

Subject	52 (train:test = 3:1)
Expression	6
Format	(channel, frame, height, weight) = (3, 8, 64, 64)
Batch size	128
Training Epochs	5000
Optimization	Adam (α=0.001, $\beta_{1}=0.9$, $\beta_{2}=0.999$)
Dimension of $z_{t}$	128
Dimension of $z_{f}$	128

**Table 3:** Root-mean-squared error for reconstruction task.

(**a**) Sprites	
	RMSE
DSVAE [14]	$5.4\times 10^{-3}\pm 0.00015$
Ours	$5.4\times 10^{-3}\pm 0.00021$
(**b**) MUG	
	RMSE
DSVAE [14]	$2.4\times 10^{-2}\pm 0.00091$
Ours	$2.7\times 10^{-2}\pm 0.00095$

**Table 4:** Average classification accuracy over ten runs (%) for the fixed static latent variable and randomly sampled dynamic latent variables.

(**a**) Sprites		
	Static	Dynamic
DSVAE [14]	**100.0** ± 0.0	100.0 ± 0.0
Ours	99.0 ± 0.2	**11.3** ± 0.4
Ground Truth	100.0	-
Random Accuracy	-	11.1
(**b**) MUG		
	Static ↑	Dynamic
DSVAE [14]	**100.0** ± 0.0	99.4 ± 0.1
Ours	99.9 ± 0.1	**21.1** ± 1.0
Ground Truth	100.0	-
Random Accuracy	-	16.7

**Table 5:** Classification accuracy (%) for the fixed dynamic latent variables and randomly sampled static latent variables. For C-DSVAE (reproduced) and ours, the average classification accuracy over ten runs is presented; the others are taken from the literature [14,15,16,17].

(**a**) Sprites		
	Static	Dynamic
DSVAE [14]	-	90.73
S3VAE [15]	-	99.49
R-WAE [16]	-	98.98
C-DSVAE [17]	-	99.99
C-DSVAE (reproduced)	**16.55** ± 0.36	**100.0** ± 0.0
Ours	16.47 ± 0.13	**100.0** ± 0.0
Ground Truth	-	100.0
Random Accuracy	16.67	-
(**b**) MUG		
	Static	Dynamic
DSVAE [14]	-	54.29
S3VAE [15]	-	70.51
R-WAE [16]	-	71.25
C-DSVAE [17]	-	**81.16**
C-DSVAE (reproduced)	**2.46** ± 0.34	47.03 ± 0.97
Ours	2.85 ± 0.69	77.52 ± 0.90
Ground Truth	-	100.0
Random Accuracy	1.92	-

**Table 6:** Entropy-based metrics for the fixed dynamic latent variable and randomly sampled static latent variable. For C-DSVAE (reproduced) and our method, the average classification accuracies over ten runs are presented; the others are taken from the literature [14,15,16,17]. In the tables, the upward (downward) arrows ↑ (↓) indicate that the higher (lower) the values, the better.

(**a**) Sprites			
	IS↑	H(y|x)↓	H(y)↑
DSVAE [14]	8.384	0.072	2.192
S3VAE [15]	8.637	0.041	**2.197**
R-WAE [16]	8.516	0.055	**2.197**
C-DSVAE [17]	8.637	0.041	**2.197**
C-DSVAE (reproduced)	**8.999** ± 0.000	**0.0001** ± 0.0000	**2.197** ± 0.000
Ours	8.999±0.000	0.0001±0.0000	2.197±0.000
Ground Truth	9.0	0.0	2.197
(**b**) MUG			
	IS↑	H(y|x)↓	H(y)↑
DSVAE [14]	3.608	0.374	1.657
S3VAE [15]	5.136	0.135	1.760
R-WAE [16]	5.149	0.131	1.771
C-DSVAE [17]	**5.341**	**0.092**	1.775
C-DSVAE (reproduced)	2.362 ± 0.052	0.855 ± 0.019	1.714 ± 0.006
Ours	4.229±0.048	0.335±0.012	1.777±0.003
Ground Truth	6.0	0.0	1.792

## Data Availability

The data presented in this study are available on request from the authors [14,26].

## Appendix A. Effect of Positions of the Adversarial Classifier

We experimentally evaluated the effect of the positions of the adversarial classifier in the two-stream architecture. For evaluation, we generated test videos using random sampling, as mentioned in Section 4.2.3, and calculated the classification accuracy. Table A1 and Table A2 show the results for four cases: (1) without the classifier (w/o classifier); (2) the position after the static branch (static); (3) the position after dynamic branches (dynamic); and (4) with two classifiers, one after each branch (both).

The position after the static branch (static) provided the best disentanglement performance because accuracies for static and dynamic classes were close to the ground truth and random accuracy. In the other cases, only one of the static or dynamic classes was closer to the ground truth and random accuracy. We achieved a similar result in two cases: without classifier (w/o classifier) and classifier after the dynamic branch (dynamic). We deduce that the static features frequently include dynamic features because the fixed or random static features affect the accuracy for both classes. When we placed the two classifiers after both branches (both), we obtained the second-best performance. Thus, placing the classifier after the static and dynamic branches had a positive and negative impact, respectively. Hence, placing the adversarial classifier after the static branch improved the disentanglement performance.

**Table A1:** Average classification accuracy over ten runs for fixed static features and randomly sampled dynamic features (%).

(**a**) Sprites		
	Static	Dynamic
w/o classifier	**100.0** ± 0.0	100.0 ± 0.0
static	99.0 ± 0.2	**11.3** ± 0.4
dynamic	**100.0** ± 0.0	100.0 ± 0.0
both	99.1 ± 1.7	21.9 ± 0.8
Ground Truth	100.0	-
Random Accuracy	-	11.1
(**b**) MUG		
	Static ↑	Dynamic
w/o classifier	**100.0** ± 0.0	99.4 ± 0.1
static	99.9 ± 0.1	**21.1** ± 1.0
dynamic	**100.0** ± 0.0	99.7 ± 0.2
both	**100.0** ± 0.0	99.5 ± 0.0
Ground Truth	100.0	-
Random Accuracy	-	16.7

**Table A2:** Average classification accuracy over ten runs for randomly sampled static features and fixed dynamic features (%).

(**a**) Sprites		
	Static	Dynamic
w/o classifier	16.3 ± 0.3	33.4 ± 0.7
static	16.5 ± 0.1	**100.0** ± 0.1
dynamic	**16.6** ± 0.2	33.3 ± 0.7
both	**16.6** ± 0.3	55.9 ± 0.7
Ground Truth	-	100.0
Random Accuracy	16.7	-
(**b**) MUG		
	Static	Dynamic ↑
w/o classifier	**1.95** ± 0.5	16.0 ± 1.7
static	2.85 ± 0.7	**77.5** ± 0.9
dynamic	1.85 ± 0.4	17.0 ± 0.8
both	1.69 ± 0.4	16.7 ± 1.4
Ground Truth	-	100.0
Random Accuracy	1.92	-

## Appendix B. Network Architecture

Here, we describe the network architectures of our sequential VAE in detail. The architectures had slightly different structures, depending on the datasets. For the Sprites dataset, we adopted the same network architecture as DSVAE [14]. For the MUG dataset, we built the architecture based on S3VAE [15], with a different decoder structure. Our decoder was a five-layer deconvolutional (transposed convolution) network. We set the kernel sizes to four in all layers. The strides of the first and second deconvolutional layers were four and two, respectively.

Figure 1 shows the overview of our method for encoding videos. The shared encoder $E_{s}$ was a five-layer convolutional network. The static encoder $E_{f}$ consisted of an MLP with a fully connected layer and a Leaky ReLU. The dynamic encoder $E_{t}$ consisted of a recurrent neural network with 256 hidden layers. We input videos into $E_{s}$ and extracted the features, which had 128 dimensions. A bi-directional LSTM with a 256-dimensional hidden layer used the extracted features as inputs, and the output was passed through the static branch. Next, using $E_{f}$, the mean $\mu_{f}$ and variance $\sigma_{f}^{2}$ of the normal distribution were extracted as the static latent variable. The output of the LSTM was also passed through the dynamic branch, in which we used $E_{t}$ to extract normally distributed mean $\mu_{t}$ and variance $\sigma_{t}^{2}$ for the dynamic latent variables.

## Appendix C. Entropy-Based Metrics

We used three disentanglement metrics: inception score, intra-entropy, and inter-entropy [17]. The process for calculating these metrics is explained below. We prepared the videos generated using random sampling generation in which the dynamic features $z_{1:T}$ were fixed, and static features $z_{f}$ were randomly sampled. We passed the generated videos x through the classifier and used the output p(y|x) metric for evaluation. y is the class label.

The inception score was defined based on the KL divergence as follows:

(A1) $$IS=\exp(E_{p(x)}\left[D_{\mathrm{KL}}\left(p\left(y|x\right)∥p\left(y\right)\right)\right]),$$

where $D_{\mathrm{KL}}\left(p\left(y|x\right)∥p\left(y\right)\right)$ denotes the KL divergence between the predicted label distribution p(y|x), which are the classifier outputs, and the marginal predicted label distribution p(y). When the class distribution is uniform, the marginal distribution p(y) will also be uniform. Therefore, the inception score approaches the number of classes.

Intra-entropy H(y|x) is the entropy of the distribution p(y|x), which is the classifier for evaluating outputs.

(A2) $$H\left(y|x\right)=-\sum_{y}p\left(y|x\right)\log p\left(y|x\right)$$

According to the output distribution of the classifier, only one class should have a value of one, while the others should be zero. The ground truth of the intra-entropy H(y|x) approaches 0.

Inter-entropy H(y) is the entropy of the marginal distribution p(y).

(A3) $$H\left(y\right)=-\sum_{y}p\left(y\right)\log p\left(y\right)$$

The marginal distribution p(y) indicates the probabilistic distribution of the classes in the dataset. When the class distribution is uniform, the marginal distribution p(y) will also be uniform. Therefore, the ground truth of inter-entropy H(y) approaches the upper bound depending on the class number.
